# The FLI portion of EWS/FLI contributes a transcriptional regulatory function that is distinct and separable from its DNA-binding function in Ewing sarcoma

**DOI:** 10.1038/s41388-021-01876-5

**Published:** 2021-06-18

**Authors:** Megann A. Boone, Cenny Taslim, Jesse C. Crow, Julia Selich-Anderson, Andrea K. Byrum, Iftekhar A. Showpnil, Benjamin D. Sunkel, Meng Wang, Benjamin Z. Stanton, Emily R. Theisen, Stephen L. Lessnick

**Affiliations:** 1 Biomedical Sciences Graduate Program, The Ohio State University, Columbus, OH USA; 2 Center for Childhood Cancer and Blood Diseases, Abigail Wexner Research Institute at Nationwide Children’s Hospital, Columbus, OH USA; 3 Molecular, Cellular, and Developmental Biology Graduate Program, The Ohio State University, Columbus, OH USA; 4 Department of Pediatrics, The Ohio State University, Columbus, OH USA

**Keywords:** Bone cancer, Paediatric cancer, Sarcoma, Mechanisms of disease, Transcription

## Abstract

Ewing sarcoma is an aggressive bone cancer of children and young adults defined by the presence of a chromosomal translocation: t(11;22)(q24;q12). The encoded protein, EWS/FLI, fuses the amino-terminal domain of EWS to the carboxyl-terminus of FLI. The EWS portion is an intrinsically disordered transcriptional regulatory domain, while the FLI portion contains an ETS DNA-binding domain and two flanking regions of unknown function. Early studies using non-Ewing sarcoma models provided conflicting information on the roles of each domain of FLI in EWS/FLI oncogenic function. We therefore sought to define the specific contributions of each FLI domain to EWS/FLI activity in a well-validated Ewing sarcoma model and, in doing so, to better understand Ewing sarcoma development mediated by the fusion protein. We analyzed a series of engineered EWS/FLI mutants with alterations in the FLI portion using a variety of assays. Fluorescence anisotropy, CUT&RUN, and ATAC-sequencing experiments revealed that the isolated ETS domain is sufficient to maintain the normal DNA-binding and chromatin accessibility function of EWS/FLI. In contrast, RNA-sequencing and soft agar colony formation assays revealed that the ETS domain alone was insufficient for transcriptional regulatory and oncogenic transformation functions of the fusion protein. We found that an additional alpha-helix immediately downstream of the ETS domain is required for full transcriptional regulation and EWS/FLI-mediated oncogenesis. These data demonstrate a previously unknown role for FLI in transcriptional regulation that is distinct from its DNA-binding activity. This activity is critical for the cancer-causing function of EWS/FLI and may lead to novel therapeutic approaches.

## Introduction

Ewing sarcoma is a bone-tumor of children and young adults [1]. These tumors contain chromosomal translocations that encode fusions between members of the FET and ETS protein families [2, 3]. In ~85% of patients, this translocation occurs at t(11;22)(q24;q12), fusing *EWSR1* to *FLI1* and effectively encoding the EWS/FLI protein [2–6]. Numerous studies have demonstrated that EWS/FLI has oncogenic function and serves as the driver of Ewing sarcoma [2, 4, 7]. Indeed, EWS/FLI is often the only genetic abnormality in these otherwise “genomically-quiet” tumors [8]. Thus, determining the mechanisms underlying the oncogenic function of EWS/FLI is critical to understanding Ewing sarcoma tumorigenesis, identifying new therapeutic approaches, and may also shed light on the oncogenic mechanisms of other “ETS-associated” tumors.

EWS/FLI functions as an aberrant transcription factor that dysregulates several thousand genes [9, 10]. EWS contributes strong transcriptional activating and repressing functions to the fusion [11–13]. The mechanisms by which the EWS-portion mediates these functions are only beginning to be understood, but include the recruitment of epigenetic co-regulators and RNA-polymerase II, perhaps via the formation of transcriptional “hubs”, phase-separated droplets, or even polymerized fibrils [9, 14–17].

FLI is a member of the ETS transcription factor family [18–20]. The ETS family is defined by the presence of highly conserved winged helix–turn–helix DNA-binding domains (DBD) [18]. The preferred high-affinity (HA) binding sequence for FLI is “ACCGGAAGTG”, while other family members bind similar sequences containing a “GGA(A/T)” core surrounded by additional base pairs [18, 21]. In addition to binding classic ETS HA sites, EWS/FLI gains the neomorphic ability to bind microsatellite sequences consisting of multiple “GGAA” repeats [22–24]. Thousands of GGAA-microsatellite sequences are scattered throughout the human genome, many of which serve as EWS/FLI-response elements associated with genes critical for Ewing sarcomagenesis [22–24]. Along with the ETS DNA-binding domain, the FLI portion of the fusion contains additional amino-terminal and carboxyl-terminal regions of uncertain function.

The cell of origin of Ewing sarcoma is unknown [25]. Early studies of the FLI portion of EWS/FLI used heterologous cell types, such as NIH3T3 murine fibroblasts, with conflicting results [25]. For example, May et al. found that expression of EWS/FLI induced oncogenic transformation of NIH3T3 cells in a manner dependent on the FLI DNA-binding domain [7]. In contrast, Welford et al. showed the DNA-binding domain of FLI was not required for EWS/FLI-mediated oncogenic transformation [26]. Subsequent studies in patient-derived Ewing sarcoma cells showed that a DNA-binding defective mutant of EWS/FLI was unable to mediate oncogenic transformation, demonstrating that DNA-binding is absolutely required for EWS/FLI-mediated transformation in a more relevant Ewing cellular model [13]. The carboxyl-terminal region of FLI (outside of the DNA-binding domain) was also evaluated in the NIH3T3 model and determined to be important for transcriptional control and oncogenic transformation mediated by EWS/FLI, though this has not been tested in a Ewing sarcoma model [27]. Furthermore, gene expression patterns mediated by EWS/FLI in the NIH3T3 model were drastically different from those in Ewing sarcoma cellular models, suggesting that EWS/FLI may utilize alternative mechanisms to drive oncogenesis in different systems and that model system selection is important [25]. To date, a systematic evaluation of the FLI portion of EWS/FLI in Ewing sarcoma cells has not been reported and so the roles of various regions of FLI in EWS/FLI-mediated oncogenic transformation remain unknown.

To address this, we analyzed the FLI portion of EWS/FLI in Ewing sarcoma cells using molecular and genomic techniques in our well-validated “knock-down/rescue” system. This model allowed us to identify a previously uncharacterized region just outside of the FLI DNA-binding domain as essential for EWS/FLI function. Mechanistic studies demonstrate a unique contribution of this region in mediating gene expression and subsequent oncogenic transformation that is independent of DNA-binding or the modulation of open chromatin states.

## Results

### Amino- and carboxyl-terminal regions of FLI are dispensable for EWS/FLI-mediated transcriptional activation in luciferase reporter assays

**Fig. 1:**
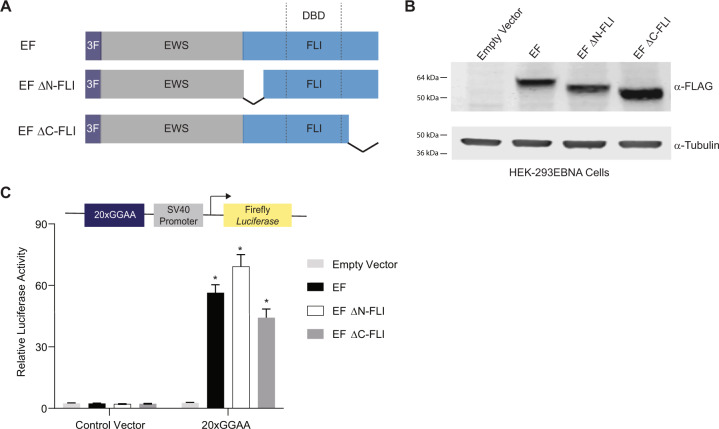
Amino- and carboxyl-terminal regions of FLI are not required for EWS/FLI-mediated transcriptional activation. **A** Protein schematic of 3xFLAG-tagged (3 F) EWS/FLI (EF) cDNA constructs. EWS is represented in gray, FLI is represented in blue, and dashed lines in the FLI portion represent the 85-amino acid ETS DNA-binding domain (DBD) of FLI. In each construct, EWS is fused directly to the FLI portion, but connecting lines are shown here to represent regions of FLI that are eliminated in each construct. EF represents a full-length “type IV” EWS/FLI translocation. EF ΔN-FLI and EF ΔC-FLI indicate constructs where EWS was fused to a version of FLI with a deletion in the amino- or carboxyl-terminal region, respectively. **B** Western blot of 3xFLAG-tagged EWS/FLI protein expression in HEK-293EBNA cells. Membranes were probed with either α-FLAG or α-tubulin antibodies. **C** Dual luciferase reporter assay results for the indicated cDNA constructs co-transfected into HEK-293EBNA cells with a Control Vector harboring no GGAA-repeats, or a vector containing 20xGGAA-repeats (represented above the graph). Data are presented as mean ± SEM (*N* = 6 biological replicates with 3 technical replicates each). Asterisks indicate that the activity of EF, EF ΔN-FLI, and EF ΔC-FLI are each statistically significant when compared to Empty Vector on a 20xGGAA μSat (*p*-value < 0.05). The activity of EF ΔN-FLI and EF ΔC-FLI are not statistically different from EF on the 20xGGAA μSat (*p*-value = 0.8).

We first sought to determine the role of the amino- and carboxyl-regions of FLI in EWS/FLI-mediated transcriptional activation using a luciferase reporter assay containing a 20xGGAA-repeat microsatellite [24]. We used a “type IV-breakpoint” EWS/FLI fusion containing regions encoded by exons 1–7 of *EWSR1* fused to exons 7–9 of *FLI1* as the full-length protein with a 3xFLAG-tag [24, 28]. We also created 3xFLAG-tagged “EF ΔN-FLI” and “EF ΔC-FLI” mutants harboring deletions amino-terminal or carboxyl-terminal to the FLI DNA-binding domain, respectively (Fig. 1A) [27]. Expression plasmids encoding these proteins were co-transfected with the 20xGGAA-microsatellite luciferase reporter into HEK-293EBNA cells (Fig. 1B). We found that all three versions of EWS/FLI were capable of activating luciferase reporter gene expression to similar levels (Fig. 1C). These data demonstrate that neither the amino-terminal nor the carboxyl-terminal region of FLI is required for transcriptional activation mediated by EWS/FLI in vitro.

### Flanking regions of the DNA-binding domain of FLI are required for oncogenic function of EWS/FLI in a Ewing sarcoma cellular model

**Fig. 2:**
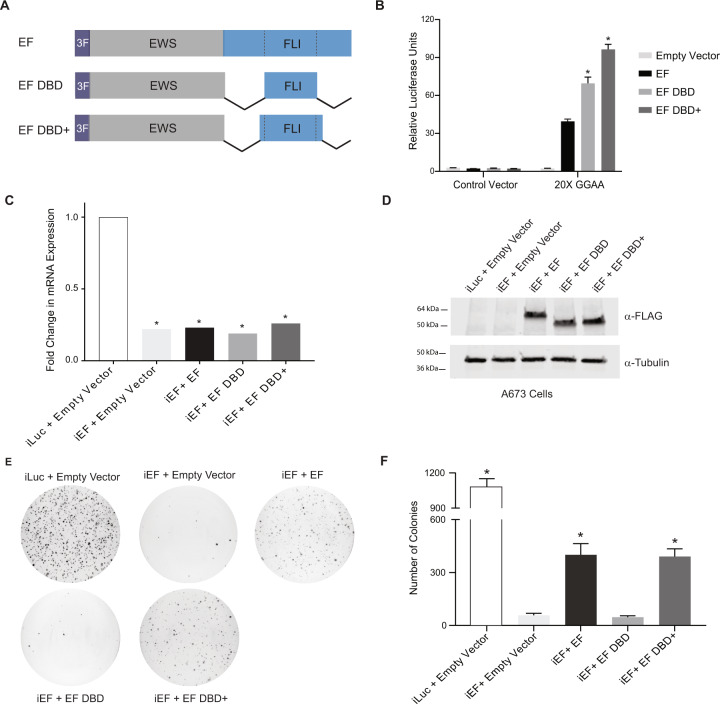
Oncogenic transformation capacity of EWS/FLI affected by short regions surrounding the FLI DBD. **A** Protein schematic of 3xFLAG-tagged (3 F) EWS/FLI cDNA constructs with deleted FLI domain regions. EF represents a full-length type IV EWS/FLI, EF DBD represents EWS fused directly to the 85-amino acid DNA-binding domain of FLI, and EF DBD+ represents EWS fused to a 102-amino acid region of FLI that contains the 85 amino-acid DNA-binding domain with 7 additional amino-acids on the amino-terminal side and 10 additional amino-acids on the carboxyl-terminal side. **B** Dual luciferase reporter assay results for the indicated constructs tested on control and 20xGGAA μSat-containing plasmids (as described in Fig. 1). Data are presented as mean ± SEM (*N* = 6 biological replicates with 3 technical replicates each). Asterisks indicate that the activity of EF DBD and EF DBD+ are each statistically higher than EF (*p*-value < 0.001). **C** Representative qRT-PCR results of endogenous EWS/FLI in A673 cells harboring the indicated constructs (iLuc is a control shRNA targeting luciferase and iEF is a shRNA targeting the 3′UTR of endogenous EWS/FLI; *N* = 1 biological replicate with 3 technical replicates for each sample). EWS/FLI mRNA values are normalized to RPL30 mRNA control values. Asterisks indicate samples are statistically different as compared to control (iLuc + Empty Vector) cells (*p*-value < 0.001). **D** Western blot analysis of exogenous EWS/FLI protein expression in the A673 knock-down/rescue cells. Protein constructs were detected using α-FLAG antibody and α-Tubulin was used as a loading control. **E** Representative soft agar assay results of A673 Ewing sarcoma cells containing the indicated constructs. **F** Soft agar assay colony formation quantification. Data presented as mean ± SEM (*N* = 9 biological replicates with 2 technical replicates each). Asterisks indicate *p*-value < 0.001 as compared to iEF + Empty Vector cells.

We next hypothesized that the only region of FLI critical for EWS/FLI activity is the ETS DNA-binding domain itself. The DNA-binding domain of FLI is not well-defined in the published literature. The ETS domain is often referred to as an 85-amino acid sequence [18, 19, 21]. However, other structural and functional studies of FLI used a larger region of FLI as the ETS domain that included short amino- and carboxyl-extensions to the 85-amino acid “core” [7, 29]. To test both “ETS domains”, we created two new mutant forms of EWS/FLI: “EF DBD” that fuses EWS directly to the 85-amino acid ETS domain and “EF DBD+” that fused EWS to a 102-amino acid ETS domain (containing 7- and 10-amino acid extensions on the amino-terminal and carboxyl-terminal sides of DBD, respectively) that has been used in prior studies (Fig. 2A) [22].

Constructs were transfected into HEK-293EBNA cells and luciferase reporter assays using the 20xGGAA-microsatellite revealed that both EF DBD and EF DBD+ induced robust transcriptional activation and were even more active than full-length EWS/FLI (EF) itself (Supplementary Fig. 1A; Fig. 2B).

To determine if the luciferase reporter results would translate to a more relevant Ewing sarcoma cellular model, we used our “knock-down/rescue” system to replace endogenous EWS/FLI with exogenous constructs in patient-derived A673 Ewing sarcoma cells [30]. Retrovirally expressed shRNAs targeting firefly luciferase (iLuc) or the 3′-UTR of endogenous EWS/FLI (iEF) were used to knock-down endogenous EWS/FLI (Fig. 2C). EWS/FLI was subsequently rescued through retroviral expression of cDNA constructs (Empty Vector, EF, EF DBD, or EF DBD+) (Fig. 2D). These cells were seeded into soft agar to measure anchorage-independent colony formation as a measure of oncogenic transformation (Fig. 2E–F). Positive control cells (iLuc + Empty Vector) showed high rates of colony formation, while cells lacking EWS/FLI (iEF + Empty Vector) showed a near total loss of transformation capacity that was rescued by re-expression of full-length EWS/FLI (iEF + EF; Fig. 2E–F). Interestingly, expression of EF DBD+ (iEF + EF DBD+) rescued colony formation to the same level as full-length EF, but the smaller EF DBD construct (iEF + EF DBD) failed to rescue colony formation (Fig. 2E–F). These data define a significant functional difference between EF DBD and EF DBD+ in the A673 Ewing sarcoma model that is not correlated to their transcriptional activity in the luciferase reporter assay.

### DNA-binding and genomic localization of EWS/FLI are nearly identical in FLI domain mutants

**Fig. 3:**
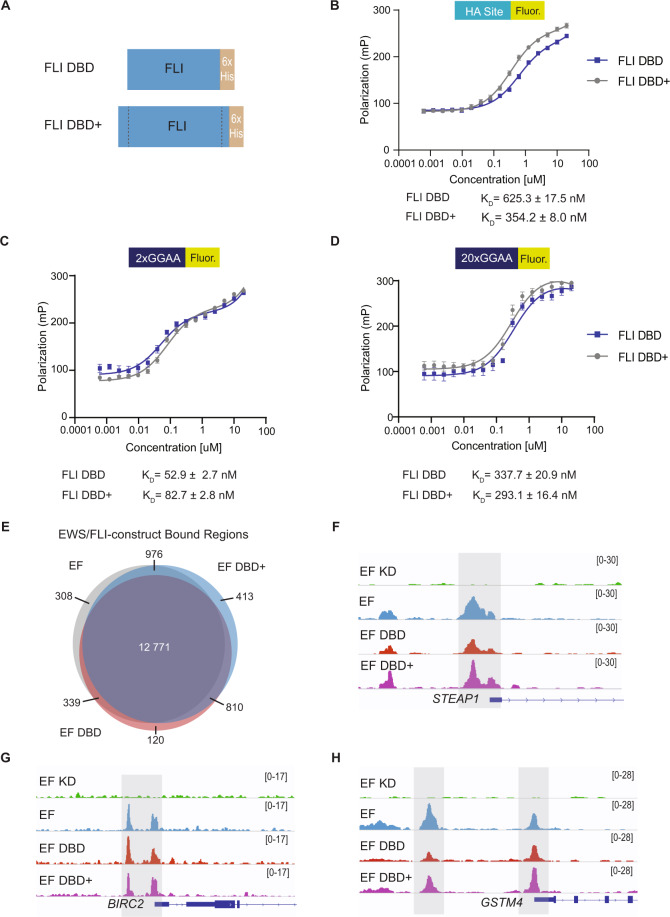
DNA-binding and genomic localization properties of EWS/FLI unaltered by deletions flanking the FLI DNA-binding domain. **A** Protein schematic of FLI DBD and FLI DBD+ recombinant protein (with C-terminal 6xHistidine-tag [6xHis]). **B**–**D** Fluorescence anisotropy assay results for FLI DBD and FLI DBD+ recombinant proteins (0–20 μM) on 5 nM fluorescein-labeled DNA sequences: **B** ETS high-affinity (HA) site DNA, **C** 2x-repeat GGAA μSat DNA, and **D** 20x-repeat GGAA μSat DNA (*N* = 2 biological replicates, 3 technical replicates each). Dissociation constants (*K*_D_) for FLI DBD and FLI DBD+ are noted for each DNA response element. **E** Venn diagram comparing peaks called in CUT&RUN for EWS/FLI construct localization in knock-down/rescue cells (EF = iEF + EF; EF DBD = iEF + EF DBD; EF DBD + = iEF + EF DBD+) when compared to cells that did not contain a rescue construct (iEF + Empty Vector) (adjusted *p*-value (FDR) < 0.05; *N* = 2 biological replicates each). The number of peaks overlapping between constructs are indicated on the Venn diagram. **F**–**H** Representative CUT&RUN peak tracks from IGV are shown for iEF + Empty Vector (EF KD), EF, EF DBD, and EF DBD+ samples. Examples of peaks from EWS/FLI-associated HA-site regulated genes ([F] *STEAP1* and [G] *BIRC2*) and GGAA-μSat-regulated genes ([H] *GSTM4*) are highlighted. Peak track scales are shown on the right.

The inability of EF DBD to rescue A673 cell colony growth suggested a loss of a critical function as compared to EF DBD+, with the only difference between the two constructs being the 17-amino acids flanking the 85-amino acid DNA-binding domain core. We therefore reasoned that these flanking amino acids may contribute to EWS/FLI DNA-binding affinity. To test this, we performed fluorescence anisotropy studies to compare the ability of FLI DBD and FLI DBD+ recombinant protein to bind fluorescein-labeled DNA (Fig. 3A, Supplementary Fig. 2A–B). We tested an ETS high-affinity (HA) site, a 2xGGAA-repeat microsatellite, and a 20xGGAA-repeat microsatellite (Fig. 3B–D). We found that both FLI DBD and FLI DBD+ bound each DNA element with similar dissociation constants (*K*_D_; Fig. 3B–D).

Although in vitro DNA-binding was similar between FLI DBD and FLI DBD + recombinant proteins, we next considered if differences in DNA-binding would be revealed in the context of a chromatinized human genome. To assess this, we performed CUT&RUN (Cleavage Under Targets & Release Under Nuclease) to determine the genomic localization of 3xFLAG-tagged EF, EF DBD, and EF DBD+ proteins in A673 cells using our knock-down/rescue system [28, 31]. An anti-FLAG antibody was used to ensure we evaluated the localization of exogenous constructs and not any low-level residual EWS/FLI remaining after knock-down. We found that CUT&RUN identified a similar number of binding peaks between EF (14 040), EF DBD+ (14 970), and EF DBD (14 394). Comparison of the binding locations for each construct demonstrated that 90% of EF DBD peaks overlap with those of EF and EF DBD+ (Fig. 3E). Further exploration of EWS/FLI-bound high-affinity sites and microsatellites did not identify any significant differences between EF DBD and EF or EF DBD+ (Fig. 3F–H). Taken together, these data indicate that there are no large-scale changes in DNA-binding capabilities that might explain the inability of EF DBD to rescue oncogenic transformation in Ewing sarcoma cells.

### EF DBD exhibits a hypomorphic gene regulatory capability in Ewing sarcoma cells

The above studies demonstrated that genome-wide localization is nearly identical between the EWS/FLI constructs. Although luciferase assays showed strong transcriptional activation by EF DBD, we considered whether the transcriptional regulatory function of EF DBD might be disrupted in a more relevant Ewing sarcoma model. To test this hypothesis, we performed RNA-sequencing on knock-down/rescue A673 cells expressing EF, EF DBD, or EF DBD+.

**Fig. 4:**
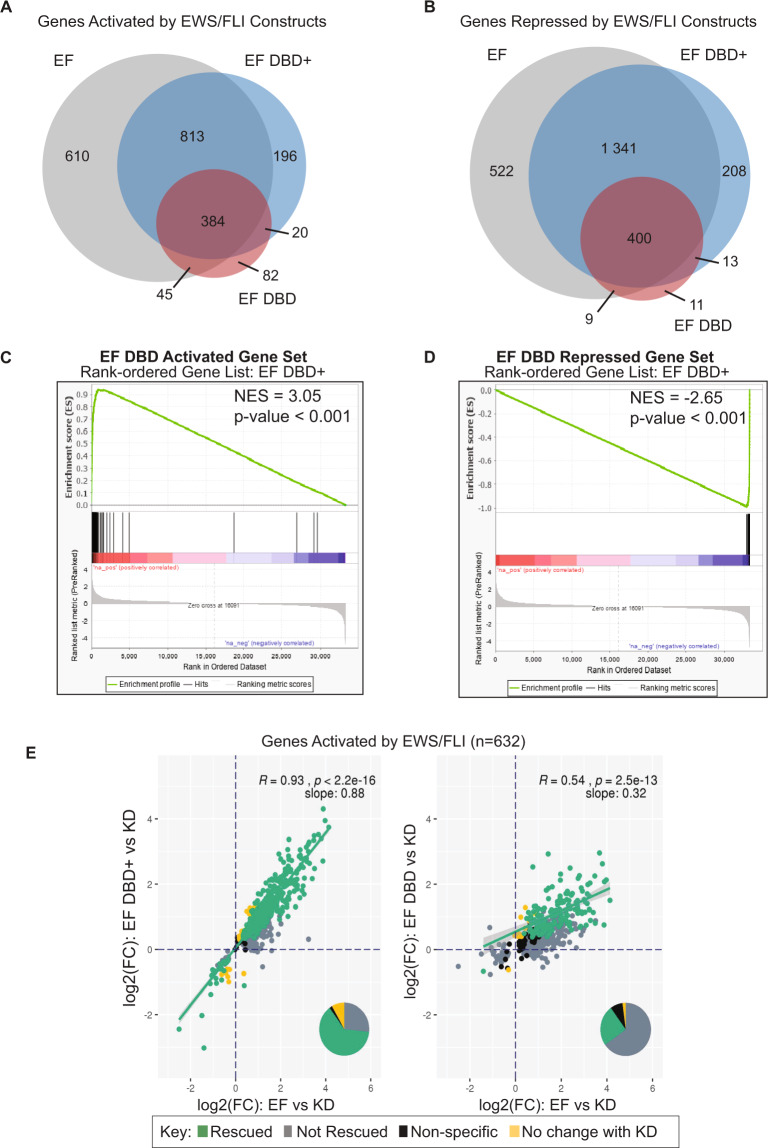
EWS/FLI-driven transcriptional regulation diminished by FLI DBD flanking deletions in Ewing sarcoma cells. **A**–**B** Venn diagram analysis of RNA-sequencing data comparing genes significantly **A** activated or **B** repressed in A673 cells rescued with the indicated constructs (full-length EWS/FLI [EF], EF DBD, and EF DBD+) when compared to A673 cells with no exogenous EWS/FLI construct (iEF + Empty Vector) (adjusted *p*-value (FDR) < 0.05; *N* = 3 biological replicates each). **C**–**D** GSEA analysis comparing all genes regulated by EF DBD+ as the rank-ordered gene list to a gene set of **C** genes activated by EF DBD (log2(FC) > 1.5, FDR < 0.05) or **D** genes repressed by EF DBD (log2(FC) < −1.5, FDR < 0.05) as the gene set. **E** Genes significantly activated by endogenous EWS/FLI were defined using a previous RNA-sequencing dataset [32]. Genes activated by EF, EF DBD, and EF DBD+ in A673 knock-down/rescue cells were compared to this list of EWS/FLI-activated genes. Scatterplots comparing genes activated by EF (on the *x*-axis) to EF DBD+ (left) or EF DBD (right) (on the *y*-axis) were plotted to determine the ability of these constructs to rescue expression these genes. Significance was defined by a log2(FC) > 0 and an adjusted *p*-value < 0.05. Pearson correlation coefficient and associated *p*-values with slope are noted on the plots. Pie charts represent the proportion of genes found in each of the described groups.

EF regulated 4124 genes and EF DBD+ regulated 3 374 genes (at adjusted *p*-values < 0.05). Importantly, 90% of the genes regulated by EF DBD+ were also regulated by EF. In contrast, EF DBD demonstrated a loss in transcriptional regulation of both activated and repressed genes, regulating only 964 genes in total (Fig. 4A–B).

We next performed a more detailed evaluation of the RNA-sequencing data using Gene Set Enrichment Analysis (GSEA). We asked where the activated and repressed gene sets of EF DBD fall in comparison to the rank-ordered gene expression list of EF DBD+. We found very strong correlations of both the activated and repressed gene sets (|NES | of 3.5 and 2.65, respectively; Fig. 4C–D). Even stronger correlations were observed when EF DBD-regulated gene sets were compared with EF activated and repressed genes (|NES | of 7.09 and 5.65; Supplementary Fig. 3A–B).

The GSEA results revealed a near-complete “stacking” of the EF DBD-regulated genes at the furthest edges of the EF DBD+ (or EF) rank-ordered lists. This suggests that EF DBD significantly rescues a portion of the EWS/FLI-regulated genes, while other genes are still regulated, but to a not statistically significant lower level. We therefore hypothesized that EF DBD functions as an attenuated, hypomorphic version of EWS/FLI. To test this hypothesis, we performed a scatterplot analysis to compare the ability of these constructs to rescue previously reported EWS/FLI-regulated genes [32]. Transcriptional regulation by EF DBD+ was highly correlated with regulation by EF for both activated (slope = 0.88, *R* = 0.93) and repressed genes (slope = 0.94, *R* = 0.97; Fig. 4E and Supplementary Fig. 3C). In contrast, EF DBD demonstrated much weaker correlations (slope = 0.32 with *R* = 0.54 for activated genes; slope = 0.54 with *R* = 0.78 for repressed genes; Fig. 4E and Supplementary Fig. 3C). These data suggest that EF DBD is regulating a similar set of genes, albeit more weakly than EF or EF DBD+.

To determine if the diminished activity of EF DBD was specific to the A673 knock-down/rescue model, we next sought to study transcriptional regulation of EF DBD and EF DBD+ in an alternative cell line. EF DBD and EF DBD+ constructs were transfected into the previously published HEK-293EBNA model system and RNA-sequencing analysis was performed (Supplementary Fig. 4A) [33]. Venn diagram analysis of significantly regulated genes for EF DBD and EF DBD+ demonstrated that a loss of activity was again observed with EF DBD, but a majority of genes regulated by EF DBD overlapped with those regulated by EF DBD+ (Supplementary Fig. 4B–C). GSEA analysis revealed a highly significant correlation of EF DBD-activated and repressed genes when compared to EF DBD+ -regulated genes (|NES| = 1.78 and 2.90, respectively; Supplementary Fig. 4D–E).

Taken together, these data indicate that EF DBD is significantly attenuated in its ability to regulate expression in multiple cell types. Thus, EF DBD is best considered a transcriptional regulatory hypomorph, even though its DNA-binding function is intact. The loss of oncogenic potential of EF DBD appears to be due to an underlying defect in transcriptional regulatory capability. This is an unanticipated result, as the transcriptional regulation function of EWS/FLI was believed to be mediated solely by the EWS-portion of the fusion with the FLI-portion contributing only DNA-binding function.

### Capacity of EWS/FLI to mediate chromatin state is unaltered by deletions surrounding the FLI DNA-binding domain

**Fig. 5:**
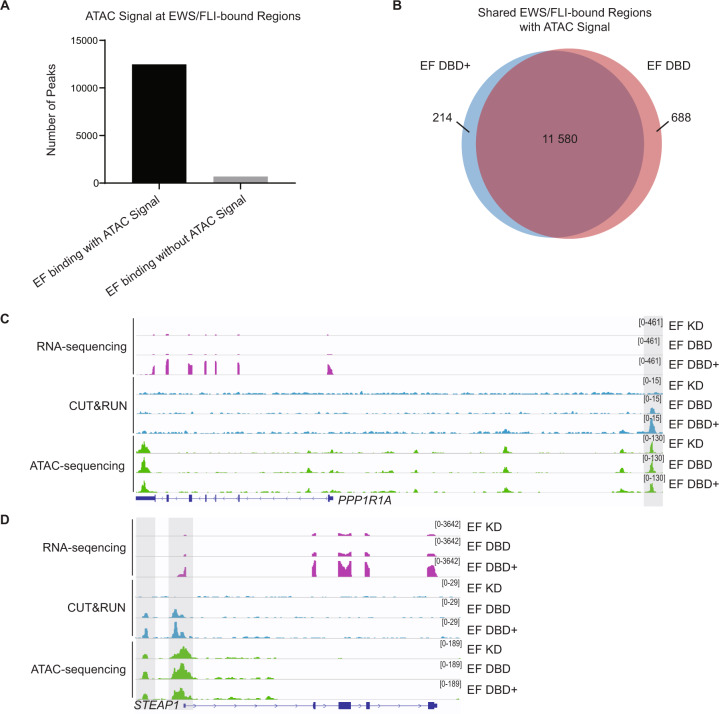
Chromatin-opening ability of EWS/FLI is unaltered by deletions flanking the FLI DNA-binding domain. **A** All EWS/FLI-bound loci in A673 cells (determined by CUT&RUN of knock-down/rescue cells expressing full-length EWS/FLI [EF]) were compared to loci harboring ATAC signal peaks and shown in graphical format (ATAC performed on *N* = 2 biological replicates each). There were 12 482 EF-bound peaks with ATAC signal and 696 EF-bound peaks without ATAC signal. **B** Venn diagram analysis of regions bound by EF DBD+ and/or EF DBD that also had overlapping ATAC signals. **C**–**D** Representative tracks of RNA-sequencing, CUT&RUN genomic localization, and ATAC-sequencing signals for the indicated knock-down/rescue A673 cells (EF KD = iEF + EF; EF DBD = iEF + EF DBD; EF DBD + = iEF + EF DBD+). Scales to view tracks were kept consistent across sequencing type in each panel and are represented on the right. Representative genes *PPP1R1A* (**C**) and *STEAP1* (**D**) are regulated by EF DBD+ but not EF DBD (adjusted *p*-value < 0.05) and overlapping CUT&RUN and ATAC-sequencing peaks are highlighted.

It was recently reported that EWS/FLI functions as a pioneer transcription factor to open regions of chromatin that were previously closed [9, 15]. As chromatin accessibility is a general necessity for transcriptional regulation, we next evaluated the role of EWS/FLI and its mutants on creation (or maintenance) of open chromatin states by performing ATAC-sequencing in our knock-down/rescue system. To focus on the role of the EWS/FLI mutants on chromatin accessibility, we overlapped EWS/FLI-bound DNA regions (identified in our CUT&RUN analysis) with the ATAC-sequencing data. We found that ~95% of the nearly 13 000 EWS/FLI-bound sites had detectable ATAC signal (Fig. 5A), indicating that most EWS/FLI binding peaks are associated with open chromatin states.

To determine if EF DBD is defective at opening chromatin, we compared the ATAC signal at regions bound by EF DBD and those bound by EF DBD+ . We found that almost 95% of ATAC peaks were shared between the two (Fig. 5B), suggesting that there were not significant differences in EWS/FLI-associated accessible chromatin in EF DBD-containing cells.

To determine if more subtle differences in open chromatin might be associated with the capability of each mutant to regulate gene expression, we performed a heatmap analysis (Supplementary Fig. 5A–B). At EWS/FLI-bound loci near genes regulated by EF DBD+ , we found that ATAC signal was similar between cells, regardless if EF DBD regulated the same genes or not. We also noted that the ATAC signal was similar at these sites in EWS/FLI knock-down cells (EF KD), indicating that the loss of EWS/FLI is not always associated with a closing of the open chromatin state, at least in this system (Fig. 5C–D). These data indicate that the dysfunction of EF DBD in mediating gene regulation is not a consequence of altered pioneer-type function to induce or maintain an open chromatin state at regulated genes.

### A fourth alpha-helix of the FLI DNA-binding domain is essential for EWS/FLI-mediated oncogenic transformation

Finally, we sought to determine which flanking region of EF DBD+ is critical for its oncogenic transformation function. We first engineered FLI DBD+ ΔN and ΔC recombinant proteins harboring deletions of either the amino-terminal 7-amino acids or the carboxyl-terminal 10-amino acids surrounding the core 85-amino acid FLI DNA-binding domain (Supplementary Fig. 6A–B). Fluorescence anisotropy performed on HA site, 2xGGAA-repeat microsatellite, and 20xGGAA-repeat microsatellite DNA revealed generally similar DNA-binding affinities with slight differences for each construct on each target DNA (Supplementary Fig. 6C–F).

**Fig. 6:**
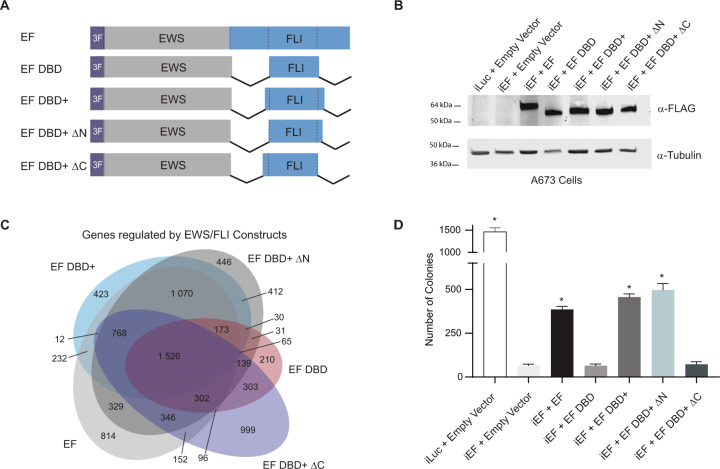
The carboxyl-terminal amino acids flanking the FLI DNA-binding domain are essential for EWS/FLI-mediated oncogenic transformation. **A** Protein schematics of 3xFLAG-tagged (3 F) EWS/FLI constructs: EF, EF DBD and EF DBD+ are described in Fig. 2. EF DBD+ ΔN represents an EWS/FLI mutant where EWS is fused to the DBD+ version of FLI missing the 7amino-terminal amino acids to the DNA-binding domain; EF DBD+ ΔC represents an EWS/FLI mutant where EWS is fused to the DBD+ version of FLI missing the 10 carboxyl-terminal amino acids to the DNA-binding domain. **B** Western blot analysis of constructs expressed in A673 cells using our knock-down/rescue system. **C** Venn diagram overlap analysis of RNA-sequencing results (*N* = 3 biological replicates each). Overlap depicts genes called as significantly regulated by the listed construct compared to control cells (iEF + Empty Vector Cells). Genes were called as significantly regulated using an FDR cut-off of 0.05 (*p*-value of overlap < 2.2e−16). **D** Soft agar assay colony formation quantification of A673 knock-down/rescue cells containing the listed knock-down and rescue constructs. Data represented by mean ± SEM (*N* = 3 biological replicates with 2 technical replicates each). Asterisks indicate *p*-value < 0.05 as compared to negative control iEF + Empty Vector sample with no EWS/FLI expression.

To study the role of the flanking regions of the FLI DNA-binding domain on EWS/FLI activity in the A673 knock-down/rescue system, we created EF DBD+ constructs harboring the same deletions as described above (EF DBD+ ΔN or EF DBD+ ΔC; Fig. 6A–B). RNA-sequencing revealed that while the EF DBD+ ΔN protein retained transcriptional regulation activity similar to EF and EF DBD+ , the EF DBD and EF DBD+ ΔC proteins showed a similar loss of regulatory capacity (Fig. 6C). This loss in transcriptional regulation correlated with oncogenic transformation capacity. Soft agar assays demonstrated that EF DBD+ ΔN was fully functional, while EF DBD+ ΔC lost the ability to transform A673 cells (Fig. 6D). These results define the 10-amino acids downstream of the FLI DNA-binding domain as essential for EWS/FLI-mediated transcriptional regulation and oncogenic transformation.

Analysis of a previously published FLI protein crystal structure revealed that this 10-amino acid sequence forms an additional fourth alpha-helix immediately downstream of the DNA-binding domain of FLI [29]. To determine if this structure is necessary for EWS/FLI-driven oncogenic transformation, we created several amino-acid mutations to disrupt the α_4_-helix of the EF DBD+ ΔN construct, which contains the smallest amount of FLI determined to retain full protein activity (EF DBD+ ΔN α-helix Mutant and EF DBD+ ΔN α-helix Pro Mutant; Supplementary Fig. 7A). Like EF DBD+ ΔC, these constructs failed to induce colony formation in A673 cells (Supplementary Fig. 7B–E). This suggests that the alpha-helix immediately downstream of the FLI DNA-binding domain is indeed contributing a critical function required for transcriptional regulation and oncogenic transformation properties of EWS/FLI.

A recent study demonstrated that recombinant FLI dimerizes via interactions between the α_4_-helix of one FLI molecule with the a_1_-helix of another FLI molecule [29]. We found that introduction of an F362A mutation, shown to disrupt FLI homodimerization, to our EF DBD+ construct had no effect on oncogenic transformation in A673 cells (Supplementary Fig. 8A–D). This suggests that homodimerization is not required for the oncogenic potential of EWS/FLI and this alpha-helical region must act in other capacities.

## Discussion

Although several studies have suggested that the regions outside of the DNA-binding domain of FLI may be important for overall EWS/FLI function, the FLI-portion of the fusion has largely been viewed as simply contributing DNA-binding function. In the current study, we took a systematic approach to understand the contributions of FLI to EWS/FLI activity in a Ewing sarcoma cellular background. This allowed us to define a previously unappreciated role for the fourth alpha-helix of the extended FLI DNA-binding domain in transcriptional regulation. This alpha-helix does not appear to be important for the DNA-binding, genomic localization, or chromatin accessibility functions of EWS/FLI. Instead, loss of this helix results in a significant loss of gene-regulatory function that culminates in a complete loss of oncogenic transformation mediated by EWS/FLI.

The mechanism(s) by which the fourth alpha-helix participates in gene regulation will require additional studies. One possibility is this fourth alpha-helix is involved in protein–protein interactions with adjacent transcription factors. Several transcription factors interact with the FLI portion of EWS/FLI, including SRF and AP-1 members that form ternary complexes with EWS/FLI on DNA [34, 35]. Published interaction sites for these factors do not map to this critical alpha-helical region and so do not readily explain the differences in activity observed between EF DBD and EF DBD+ proteins. EWS/FLI may interact with other transcription factors via this region; however, we do not favor a loss of such EWS/FLI-transcription factor interactions as the most likely cause of the massive loss of transcriptional function by EF DBD. We reason that if there were losses of EWS/FLI interactions with specific transcription factors, we may have expected a more limited loss of gene expression (rather than the ~70% loss observed for EF DBD). Furthermore, the formation of ternary complexes between pairs of transcription factors with DNA tend to stabilize DNA binding, so we might also have anticipated a significant change in genomic localization of EF DBD, which was not observed. We currently favor a model whereby the fourth alpha-helix interacts with epigenetic regulators and/or components of the core transcriptional machinery that are required for global gene regulation, rather than regulation limited to specific loci.

Work in NIH3T3 murine fibroblasts suggested a role for the carboxyl-terminal region of FLI in mediating transcriptional downregulation by EWS/FLI [27]. Our work here rules out a significant role for this region in EWS/FLI-mediated oncogenesis. Additionally, luciferase reporter assays have long been used as functional screens, but our results demonstrate that activation on a luciferase reporter does not necessarily reflect function in a Ewing sarcoma cellular background. Indeed, we also note that we did not see direct evidence of the pioneer-type function of EWS/FLI in the Ewing sarcoma model, which had been previously observed in a mesenchymal stem cell model [9]. In our system, EWS/FLI-occupied sites remained open and accessible following knock-down of EWS/FLI. It may be that the 80–90% knock-down we achieved was insufficient to allow for chromatin closing of those loci or perhaps insufficient time was provided to allow for chromatin closing. Nevertheless, changes in chromatin accessibility were not associated with the transcriptional dysfunction exhibited by EF DBD. These findings highlight the importance of analyzing EWS/FLI activity in a relevant Ewing sarcoma cellular context.

A detailed comparison of ETS protein structures revealed that many harbor this additional fourth alpha-helix downstream of their DNA-binding domains. As such, the work presented here may have relevance beyond an EWS/FLI context. For example, Ewing sarcoma translocations involve one of five closely homologous ETS family members (FLI, ERG, FEV, ETV1, and ETV4) [11]. Additionally, *TMPRSS2-ERG* fusions exist in approximately 50% of prostate cancer cases, with *TMPRSS2-FEV*, *-ETV1*, *-ETV4*, and *-ETV5* fusions found in other patients [36]. In fact, ETS family members have been implicated in numerous solid and liquid tumors via overexpression, amplification, mutations, and translocations [20]. As the functional motif we identified as crucial for EWS/FLI activity is conserved in numerous ETS factors, the data presented in this report may have wide-ranging implications for oncogenesis in multiple tumor types.

In summary, we have taken a systematic structure–function approach to identify a previously unappreciated region in the extended FLI DNA-binding domain that is required for transcriptional regulation and oncogenic transformation mediated by EWS/FLI. This transcriptional function is distinct from the DNA-binding and genomic localization functions typically associated with the ETS domain. This work has implications not only for the development of Ewing sarcoma, but may also be useful in understanding the development of other ETS-associated tumors and, perhaps, even normal ETS transcriptional function. A better understanding of this newly defined region may lead to novel approaches for therapeutically targeting EWS/FLI, as well as other ETS factors.

## Materials and methods

### Constructs and retroviruses

Puromycin-resistant retroviral vectors encoding shRNAs targeting Luciferase (iLuc; sequence: 5′-GATCCCCCTTACGCTGAGTACTTCGATTCAAGAGATCGAAGTACTCAGCGTAAGTTTTTGGAAC-3”) or the 3′-UTR of endogenous EWS/FLI mRNA (iEF; sequence: 5′-GATCCCCATAGAGGTGGGAAGCTTATTTCAAGAGAATAAGCTTCCCACCTCTATTTTTTGGAAC-3′) were previously described [24, 28]. Full-length EWS/FLI and mutants (all containing amino-terminal 3xFLAG-tags) were cloned into pMSCV-Hygro (Invitrogen) with sequence details provided in Supplementary Table 1. Luciferase reporter constructs (in pGL3 vectors; Promega Corporation) were previously described [24]. Recombinant proteins (with a carboxyl-terminal 6xHistidine tag) were expressed using pET28a plasmids (EMD Chemicals).

### Cell culture methods

HEK-293EBNA (Invitrogen) and A673 cells (ATCC) were grown, retroviruses produced and used for infection, and soft agar assays were performed as described [24, 28, 37]. STR profiling and mycoplasma testing are performed annually on all cell lines. Dual luciferase reporter assays were performed in HEK-293EBNA cells as previously described [24]. 3.75–5.0 microgram of cDNA constructs were transfected into HEK-293EBNA cells and collected 48 h later for RNA-sequencing analysis.

### Immunodetection

Whole-cell or nuclear protein extraction, protein quantification, and Western blot analysis was performed as previously described [24, 28, 37]. Immunoblotting was performed using anti-FLAG M2 mouse (Sigma F1804-200UG), anti-α-Tubulin (Abcam ab7291), and anti-Lamin B1 (Abcam ab133741). Membranes were imaged using the LiCor Odyssey CLx Infrared Imaging System.

### qRT-PCR

Total RNA was extracted from cells using the RNeasy Extraction Kit (Qiagen 74136). Reverse transcription and qPCR were performed using the iTaq Universal SYBR Green 1-Step Reaction Mix (BioRad 1725151) on a Bio-Rad CFX Connect Real-Time System. Primer sequences are found in Supplementary Table 2.

### Recombinant protein purification

Recombinant 6xHistidine-tagged proteins were prepared from *E.coli* BL21(DE3) cells transformed with pET28a plasmids. Cells were resuspended (25 mM Tris-HCl, pH 7.9, 1 M NaCl, 0.1 mM EDTA, 1 mM PMSF, 5 mM imidazole, proteinase inhibitors (Roche 4693159001)) and lysed via sonication. The lysate was centrifuged at 10,000 × *g* for 30 min and the supernatant incubated with Ni-NTA resin (Qiagen) for 1 h at 4 °C. Resin-bound protein was washed over a column with 90 mL of lysis buffer and eluted using lysis buffer containing 500 mM imidazole. Eluted protein was dialyzed overnight (300 mM KCl, 25 mM Bis-Tris, 0.05% sodium azide, 5 mM β-ME), treated with nuclease (Pierce 88700), and purified by ion-exchange chromatography as previously described [38]. IEC fractions were combined, dialyzed into storage buffer (10% glycerol, 65 mM KCI, 25 mM Tris-HCl-pH 7.9, 6 mM MgCl2, 0.5 mM EDTA, 0.2 mM PMSF, 1 mM DTT), and concentrated using Amicon Ultra centrifugal filter units. A260/A280 ratio for purified proteins were determined to be between 0.55 and 0.58.

### Fluorescence anisotropy

Fluorescence anisotropy was performed as previously described [24]. Recombinant protein sequences and fluorescein-labeled DNA duplex sequences (ordered from IDT) are found in Supplementary Tables 1 and 3, respectively.

### CUT&RUN and analysis

Two biological replicates for each knock-down/rescue sample were analyzed by CUT&RUN using the anti-FLAG M2 mouse antibody (Sigma F1804-200UG) as described and sequenced with the Illumina HiSeq4000 [28]. Raw reads were trimmed, de-duplicated, aligned to hg19 reference genomes, and peaks were called using macs2 and DiffBind (Bioconductor) using “iEF + Empty Vector” samples as controls [39]. Bigwig files combining two replicates with normalization option “RPGC” were created using Deeptools [40]. Overlapping peak analysis was completed using R packages ChIPpeakAnno and Genomic Ranges [41, 42].

### RNA-sequencing and analysis

RNA-sequencing was performed on three biological replicates for knock-down/rescue A673 samples in three separate experiments (Figs. 4, Supplementary Fig. 4, and Fig. 6, respectively). TruSeq Stranded mRNA Kit (Illumina Cat. No. 20020594) was used to prepare cDNA libraries from total RNA and sequenced on Illumina HiSeq4000 to generate 150-bp paired-end reads. Reads were analyzed for quality control, trimmed, aligned to the human genome and analyzed for differential analysis (using FASTQC, Multiqc, Trim_galore, STAR version 2.5.2b, DESeq2) [43]. GSEA (Version 4.0.3) analysis was performed: significantly activated and repressed genes were defined using an FDR < 0.05 cut-off for EF DBD to create gene sets. EF DBD+ or EF genes were used as the rank-ordered gene list to compare with these gene sets [44]. RNA-expression scatterplot analysis was performed as previously described [28].

### ATAC-sequencing and analysis

ATAC-sequencing was performed on two separate biological replicates for knock-down/rescue A673 cells as previously described and sequenced with Illumina HiSeq4000 [45, 46]. The ENCODE pipeline was used for trimming, alignment to hg19 reference genome, and peak calling on individual replicates (ENCODE Project). RegioneR was used to perform permutation test and test significance of overlapping ATAC peaks in different samples [42]. EnrichedHeatmap, ggplot2, ChIPpeakAnno, and GenomicRanges were used to calculate overlapping regions and create heatmaps [41, 42, 47, 48]. Differential ATAC peak analysis was completed using DiffBind (Bioconductor) and DESeq2 with an FDR < 0.05 [43].

### Statistical analysis

Luciferase assay, soft agar assay, and PCR data are presented as mean ± SEM. Fluorescence anisotropy data are presented as mean ± SEM. Significance of experimental results was determined using a two-sided Student’s *t* test for comparison between groups. *P*-values less than 0.05 were considered to be significant.

## Supplementary information

Supplementary Figures 1-8

Supplementary Tables

Supplemental Figure and Table Legends

## Data Availability

The sequencing datasets generated and analyzed during the current study are available in the Gene Expression Omnibus and accessible at GSE160898. All other data generated or analyzed during this study are available from the corresponding author on reasonable request.

## Code availability

All code used to analyze sequencing data as described above are from publically available resources.
