# Supplementary material for: The FLI portion of EWS/FLI contributes a transcriptional regulatory function that is distinct and separable from its DNA-binding function in Ewing sarcoma
Source: Oncogene. 2021 Jun 18;40(29):4759–69. doi: 10.1038/s41388-021-01876-5 (PMC8298202; doi:10.1038/s41388-021-01876-5)
Supplement: Supplementary file 1 — Supplementary Figures 1-8 [file 41388_2021_1876_MOESM1_ESM.pdf]

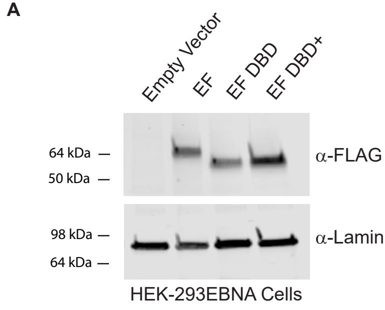
A
Empty Vector
EF
EF DBD
EF DBD+
64 kDa
50 kDa
α-FLAG
98 kDa
64 kDa
α-Lamin
HEK-293EBNA Cells

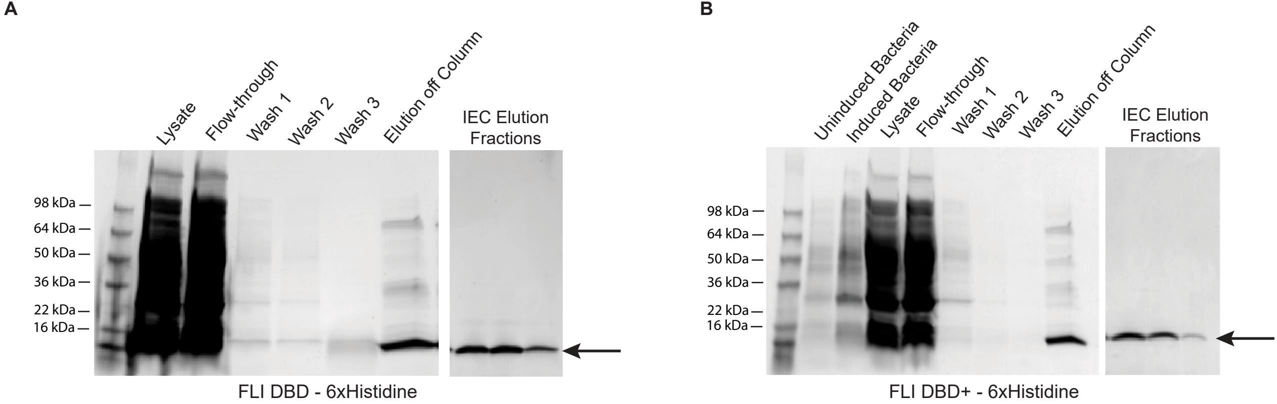
A
Lysate
Flow-through
Wash 1
Wash 2
Wash 3
Elution off Column
IEC Elution Fractions
98 kDa
64 kDa
50 kDa
36 kDa
22 kDa
16 kDa
FLI DBD - 6xHistidine
B
Uninduced Bacteria
Induced Bacteria
Lysate
Flow-through
Wash 1
Wash 2
Wash 3
Elution off Column
IEC Elution Fractions
98 kDa
64 kDa
50 kDa
36 kDa
22 kDa
16 kDa
FLI DBD+ - 6xHistidine

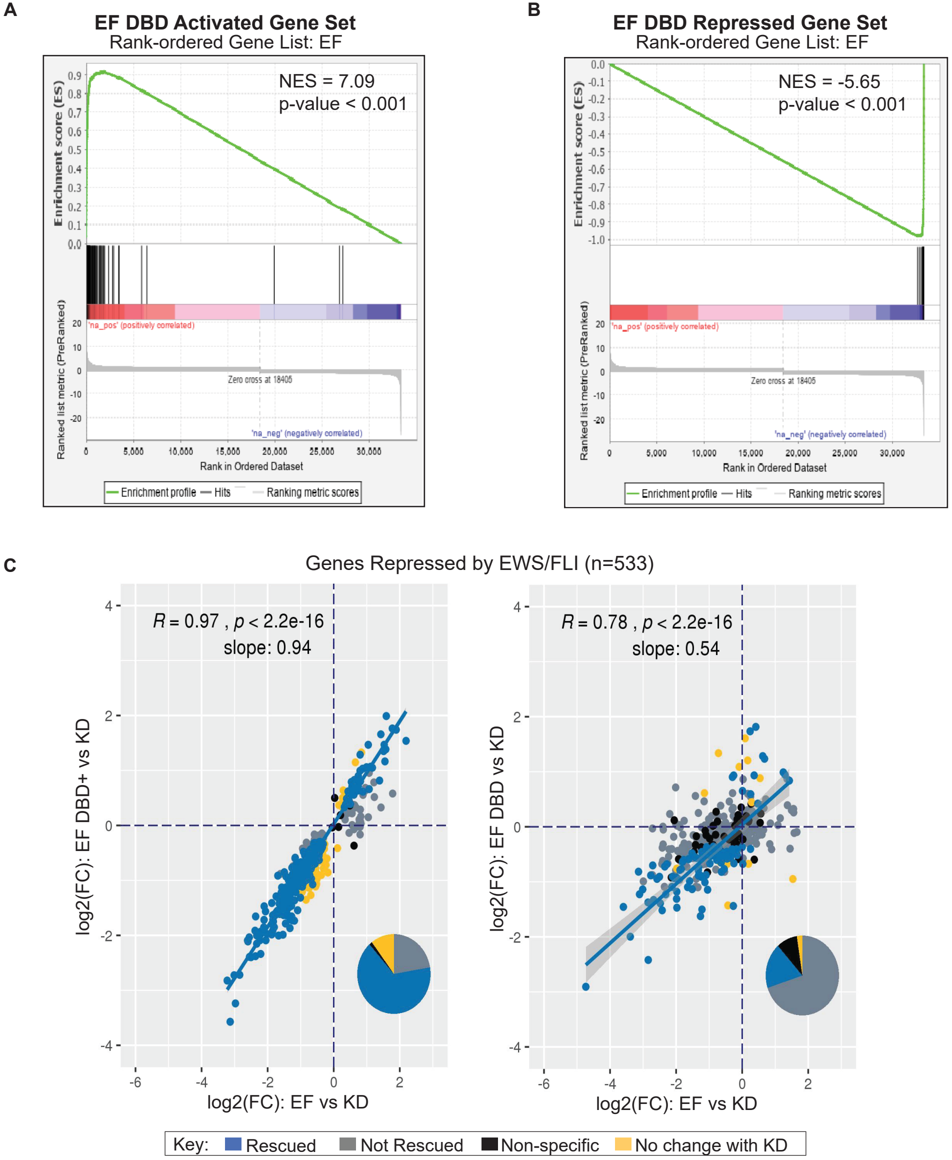
A
EF DBD Activated Gene Set
Rank-ordered Gene List: EF
NES = 7.09
p-value < 0.001
Enrichment score (ES)
Ranked list metric (PreRanked)
'na_pos' (positively correlated)
Zero cross at 18405
'na_neg' (negatively correlated)
Rank in Ordered Dataset
Enrichment profile — Hits — Ranking metric scores
B
EF DBD Repressed Gene Set
Rank-ordered Gene List: EF
NES = -5.65
p-value < 0.001
Enrichment score (ES)
Ranked list metric (PreRanked)
'na_pos' (positively correlated)
Zero cross at 18405
'na_neg' (negatively correlated)
Rank in Ordered Dataset
Enrichment profile — Hits — Ranking metric scores
C
Genes Repressed by EWS/FLI (n=533)
R = 0.97 , p < 2.2e-16
slope: 0.94
log2(FC): EF DBD+ vs KD
log2(FC): EF vs KD
R = 0.78 , p < 2.2e-16
slope: 0.54
log2(FC): EF DBD vs KD
log2(FC): EF vs KD
Key: Rescued Not Rescued Non-specific No change with KD

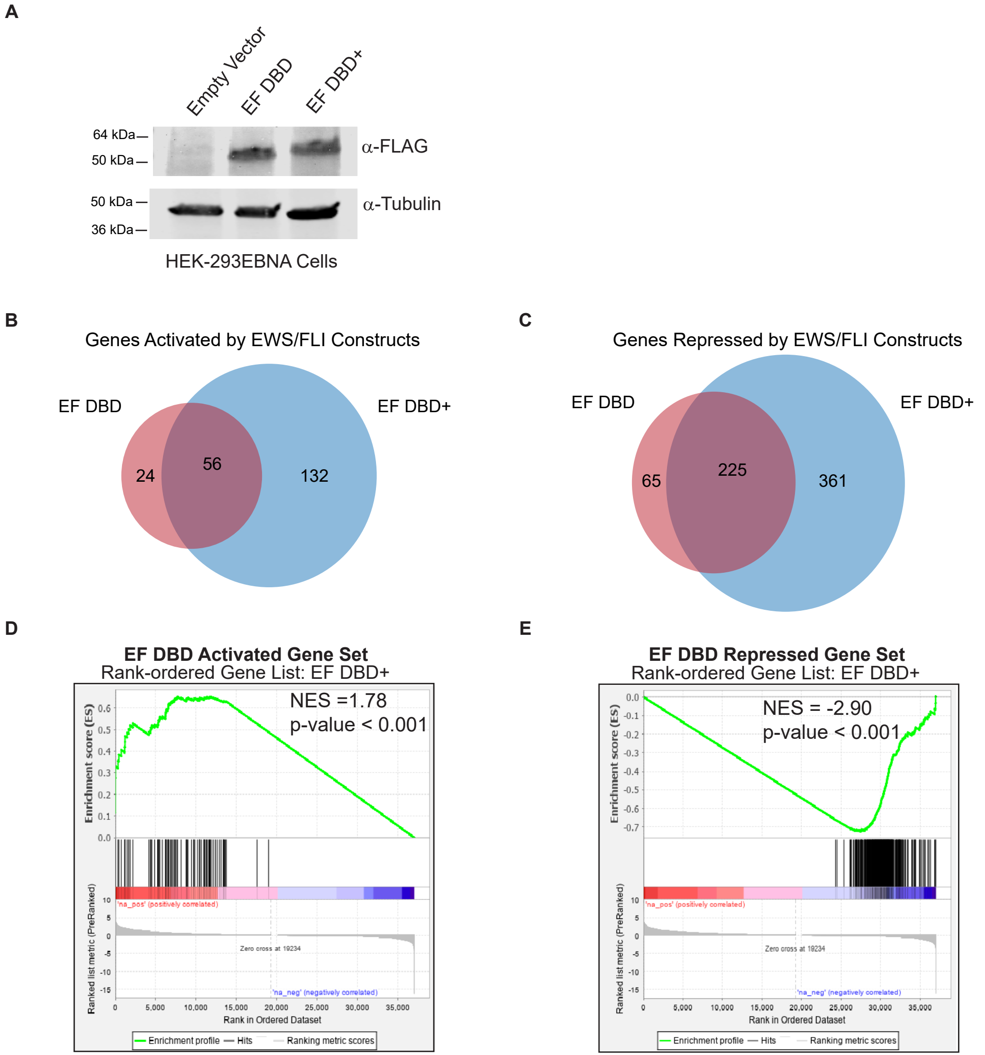
A
Empty Vector
EF DBD
EF DBD+
64 kDa
50 kDa
α-FLAG
50 kDa
36 kDa
α-Tubulin
HEK-293EBNA Cells
B
Genes Activated by EWS/FLI Constructs
EF DBD
EF DBD+
24
56
132
C
Genes Repressed by EWS/FLI Constructs
EF DBD
EF DBD+
65
225
361
D
EF DBD Activated Gene Set
Rank-ordered Gene List: EF DBD+
NES =1.78
p-value < 0.001
Enrichment score (ES)
Ranked list metric (PreRanked)
'na_pos' (positively correlated)
Zero cross at 19234
'na_neg' (negatively correlated)
Rank in Ordered Dataset
Enrichment profile
Hits
Ranking metric scores
E
EF DBD Repressed Gene Set
Rank-ordered Gene List: EF DBD+
NES = -2.90
p-value < 0.001
Enrichment score (ES)
Ranked list metric (PreRanked)
'na_pos' (positively correlated)
Zero cross at 19234
'na_neg' (negatively correlated)
Rank in Ordered Dataset
Enrichment profile
Hits
Ranking metric scores

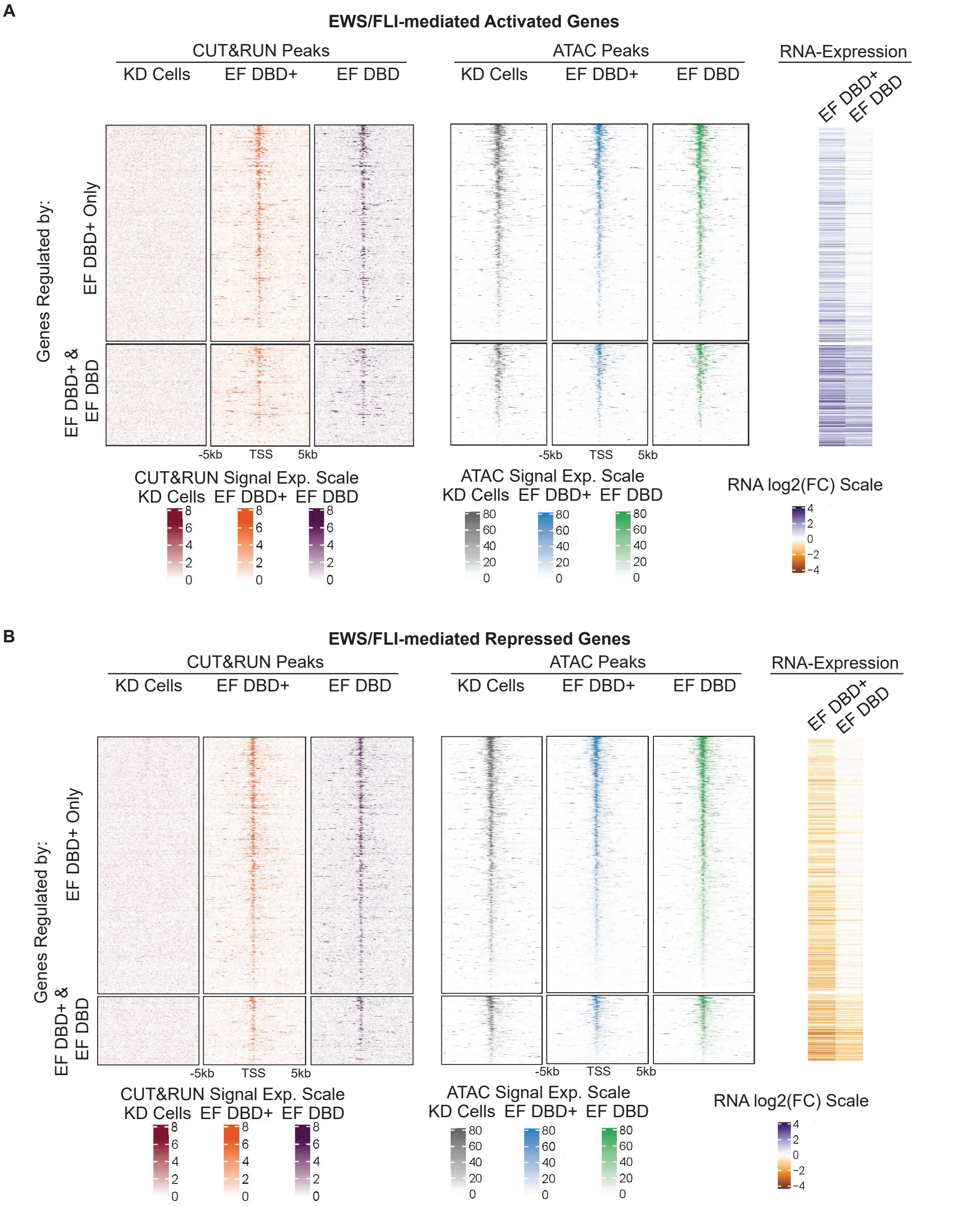
A
EWS/FLI-mediated Activated Genes
CUT&RUN Peaks
ATAC Peaks
RNA-Expression
KD Cells
EF DBD+
EF DBD
Genes Regulated by:
EF DBD+ Only
EF DBD+ & EF DBD
-5kb
TSS
5kb
CUT&RUN Signal Exp. Scale
ATAC Signal Exp. Scale
RNA log2(FC) Scale
B
EWS/FLI-mediated Repressed Genes

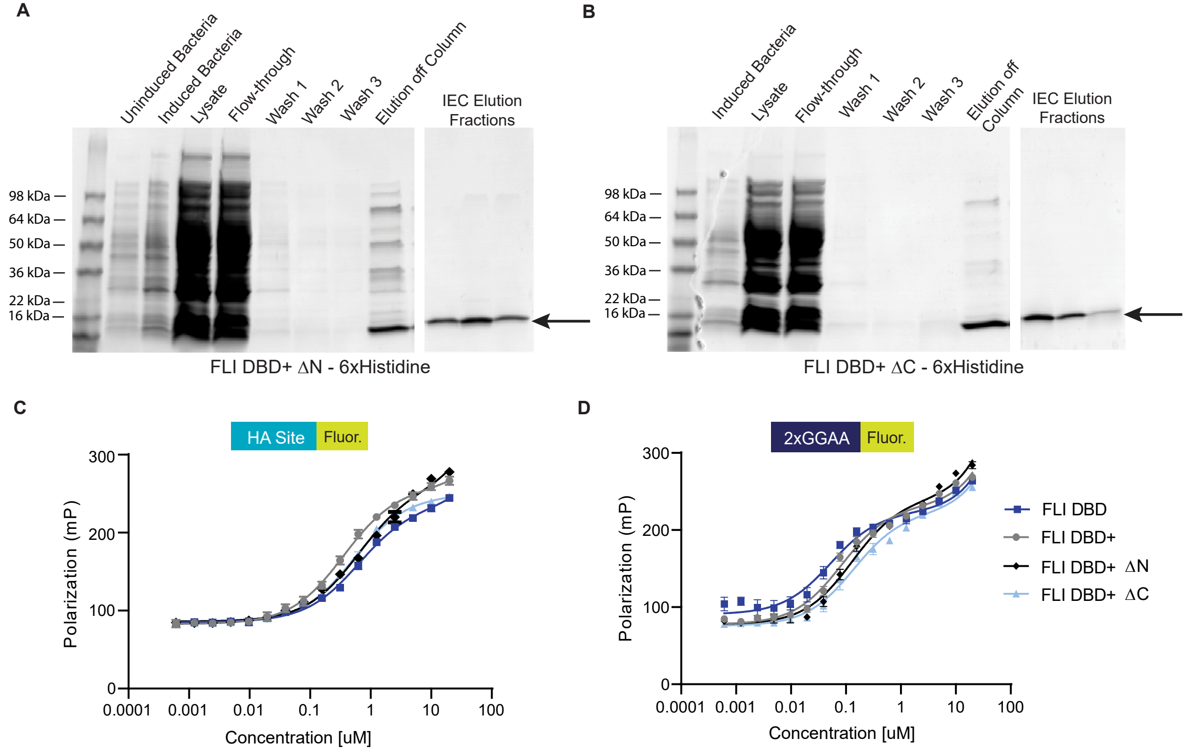

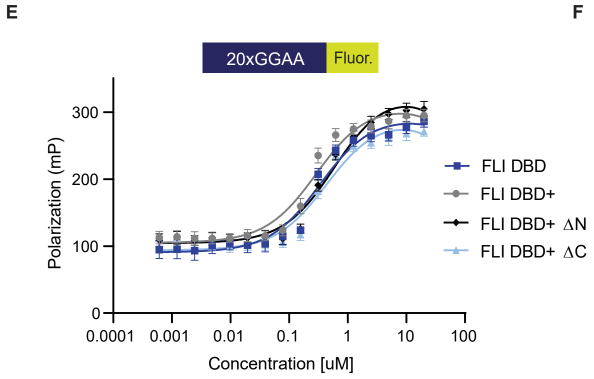

F

Construct	K_D
HA Site	
FLI DBD	K_D= 625.3 ± 17.5 nM
FLI DBD+	K_D= 354.2 ± 8.0 nM
FLI DBD+ ΔN	K_D= 616.4 ± 19.5 nM
FLI DBD+ ΔC	K_D= 517.5 ± 11.9 nM
2xGGAA	
FLI DBD	K_D= 52.9 ± 2.7 nM
FLI DBD+	K_D= 82.7 ± 2.8 nM
FLI DBD+ ΔN	K_D= 128.2 ± 4.7 nM
FLI DBD+ ΔC	K_D= 137.6 ± 5.3 nM
20xGGAA	
FLI DBD	K_D= 337.7 ± 20.9 nM
FLI DBD+	K_D= 293.1 ± 16.4 nM
FLI DBD+ ΔN	K_D= 598.6 ± 31.0 nM
FLI DBD+ ΔC	K_D= 442.5 ± 26.4 nM

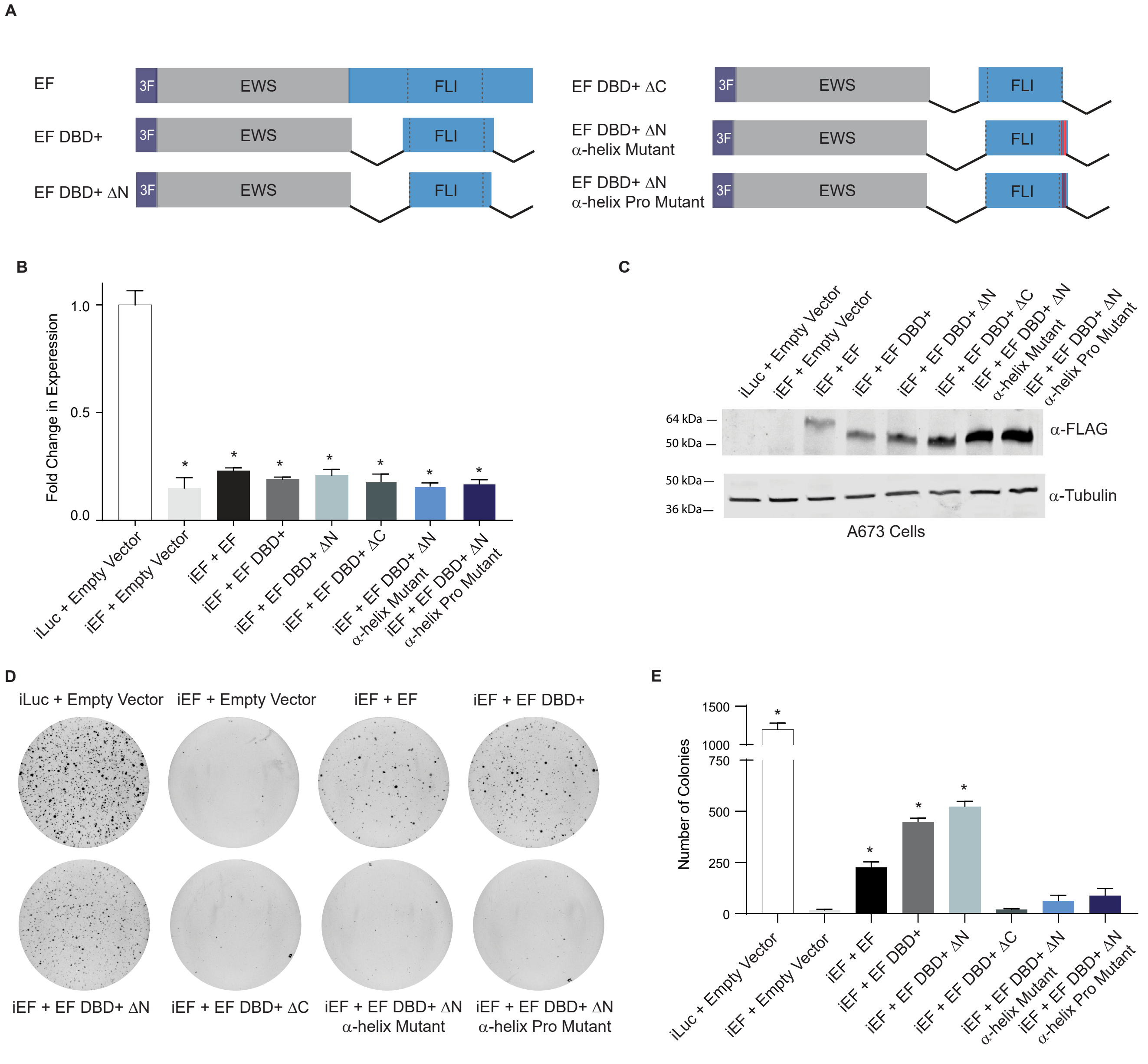
A
EF
3F
EWS
FLI
EF DBD+
EF DBD+ ΔN
EF DBD+ ΔC
EF DBD+ ΔN α-helix Mutant
EF DBD+ ΔN α-helix Pro Mutant
B
Fold Change in Experession
1.0
0.5
0.0
iLuc + Empty Vector
iEF + Empty Vector
iEF + EF
iEF + EF DBD+
iEF + EF DBD+ ΔN
iEF + EF DBD+ ΔC
iEF + EF DBD+ ΔN α-helix Mutant
iEF + EF DBD+ ΔN α-helix Pro Mutant
C
64 kDa
50 kDa
α-FLAG
50 kDa
36 kDa
α-Tubulin
A673 Cells
D
E
Number of Colonies
1500
1000
750
500
250
0

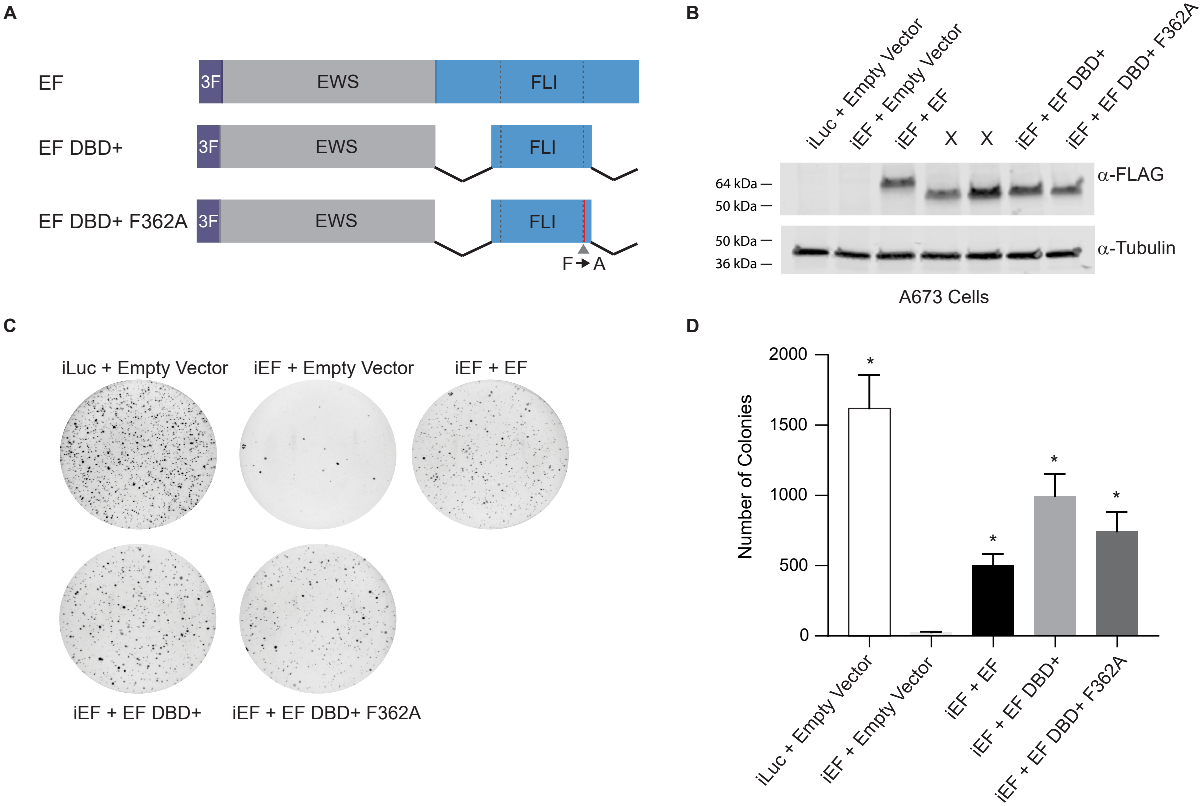

A
EF
3F
EWS
FLI
EF DBD+
3F
EWS
FLI
EF DBD+ F362A
3F
EWS
FLI
F → A
B
iLuc + Empty Vector
iEF + Empty Vector
iEF + EF
X
X
iEF + EF DBD+
iEF + EF DBD+ F362A
64 kDa
50 kDa
α-FLAG
50 kDa
36 kDa
α-Tubulin
A673 Cells
C
iLuc + Empty Vector
iEF + Empty Vector
iEF + EF
iEF + EF DBD+
iEF + EF DBD+ F362A
D
Number of Colonies
2000
1500
1000
500
0
*
*
*
*
iLuc + Empty Vector
iEF + Empty Vector
iEF + EF
iEF + EF DBD+
iEF + EF DBD+ F362A
