# Supplementary material for: The FLI portion of EWS/FLI contributes a transcriptional regulatory function that is distinct and separable from its DNA-binding function in Ewing sarcoma
Source: Oncogene. 2021 Jun 18;40(29):4759–69. doi: 10.1038/s41388-021-01876-5 (PMC8298202; doi:10.1038/s41388-021-01876-5)
Supplement: Supplementary file 2 — Supplementary Tables [file 41388_2021_1876_MOESM2_ESM.docx]

**Supplementary Table 1. Amino acids references for EWS/FLI or FLI recombinant protein-encoding constructs.**

|  | **Amino acids residues according to protein:** | | |
| --- | --- | --- | --- |
| **Construct Name** | **EWSR1**  (NP_001156757.1) | **FLI1**  (NP_002008.2) | **“Type IV” EWS/FLI**  (1) |
| Full-length “Type IV” EF | 1-265 | 242-452 | 266-476 |
| EF ΔN-FLI | 1-265 | 275-452 | 299-476 |
| EF ΔC-FLI | 1-265 | 242-373 | 266-397 |
| EF DBD | 1-265 | 277-361 | 302-386 |
| EF DBD+ | 1-265 | 270-371 | 295-396 |
| FLI DBD | - | 277-361 | 302-386 |
| FLI DBD+ | - | 270-371 | 295-396 |
| FLI DBD+ ΔN | - | 277-371 | 302-396 |
| FLI DBD+ ΔC | - | 270-361 | 295-386 |
| EF DBD+ ΔN | 1-265 | 277-371 | 302-396 |
| EF DBD+ ΔC | 1-265 | 270-361 | 295-386 |
| EF DBD+ F362A | 1-265 | 270-371, F362A | 295-396, F362A |
| EF DBD+ ΔN α-helix Mutant | 1-265 | 277-371, H363D, I365S, Q367D, L369S | 302-396, H388D, I390S, Q392D, L394S |
| EF DBD+ ΔN α-helix Pro Mutant | 1-265 | 277-371, G364P, I365P, G366P, R367P, L368P | 302-396, G389P, I390P, G391P, R392P, L393P |

**Supplementary Table 2. Sequences for primers used in qRT-PCR experiments.**

| **Gene** | **Forward Primer** | **Reverse Primer** |
| --- | --- | --- |
| EWS/FLI | 5’-CAGTCACTGCACCTCCATCC | 5’-TTCATGTTATTGCCCCAAGC |
| RPL30 | 5’-GGGGTACAAGCAGACTCTGAAG | 5’-ATGGACACCAGTTTTAGCCAAC |

**Supplementary Table 3. Fluorescein-labeled DNA-duplex oligonucleotides used for fluorescence anisotropy experiments.**

| **DNA Duplex** | **Sequence (forward strand of duplex)** |
| --- | --- |
| High Affinity (HA) Site | /56FAM/TTTACCGGAAGTGTT |
| 2X GGAA | /56FAM/TTTGGAAGGAATTT |
| 20X GGAA | /56FAM/ TTTGGAAGGAAGGAAGGAAGGAAGGAAGGAAGGAAGGAAGGAAGGAAGGAAGGAAGGAAGGAAGGAAGGAAGGAAGGAAGGAATTT |

**Supplementary References:**

1. May WA, Gishizky ML, Lessnick SL, Lunsford LB, Lewis BC, Delattre O, et al. Ewing sarcoma 11;22 translocation produces a chimeric transcription factor that requires the DNA-binding domain encoded by FLI1 for transformation. Proc Natl Acad Sci U S A. 1993;90(12):5752-6.
