# Supplementary material for: The FLI portion of EWS/FLI contributes a transcriptional regulatory function that is distinct and separable from its DNA-binding function in Ewing sarcoma
Source: Oncogene. 2021 Jun 18;40(29):4759–69. doi: 10.1038/s41388-021-01876-5 (PMC8298202; doi:10.1038/s41388-021-01876-5)
Supplement: Supplementary file 3 — Supplemental Figure and Table Legends [file 41388_2021_1876_MOESM3_ESM.docx]

**Supplementary Figure 1. EWS/FLI mutant construct expression in HEK-293EBNA cells.**

(A) 3xFLAG-tagged full-length EWS/FLI (EF), EF DBD, or EF DBD+ constructs were expressed in HEK-293EBNA cells. Western blot analysis was used to determine expression of these proteins utilizing an α-FLAG antibody. α-Lamin antibody was used as a loading control.

**Supplementary Figure 2.** **Recombinant FLI DBD and FLI DBD+ protein purification.**

(A-B) Samples were taken at several stages of recombinant protein purification for (A) FLI DBD - 6xHisitidine and (B) FLI DBD+ - 6xHistidine, including: uninduced bacteria, induced bacteria, after bacterial lysis, after wash 1, wash 2, and wash 3, eluted fraction from the column, and eluted fractions after performing ion-exchange chromatography (IEC). Samples were run on SDS-PAGE gels and visualized using Coomassie blue staining.

**Supplementary Figure 3. Deletions surrounding the FLI DBD of EWS/FLI result in weaker transcriptional regulation.**

(A-B) GSEA analysis comparing genes regulated by EF as the rank-ordered gene list to a gene set of (A) activated genes by EF DBD (log2(FC) > 1.5, FDR < 0.05) or (B) repressed genes by EF DBD (log2(FC) < -1.5, FDR < 0.05). (C) Genes significantly repressed by endogenous EWS/FLI were defined using a previous RNA-sequencing dataset (32). Genes repressed by EF, EF DBD, and EF DBD+ in A673 knock-down/rescue cells were compared to this list of EWS/FLI-repressed genes. Scatterplots comparing genes repressed by exogenous EF (on the x-axis) to EF DBD+ (left) or EF DBD (right) (on the y-axis) were plotted to determine the ability of these constructs to rescue repression of these genes. Significance was defined by a log2(FC) < 0 and an adjusted p-value < 0.05. Pearson correlation coefficient and associated p-values with slope are noted on the plots. Pie charts represent the proportion of genes found in each of the described groups.

**Supplementary Figure 4. EF DBD exhibits hypomorphic ability to drive transcription in alternative HEK-293EBNA cell model.**

(A) Western blot analysis of EF DBD and EF DBD+ cDNA-containing constructs transfected into HEK-293EBNA cells before collection for RNA-sequencing analysis. α-FLAG and α-tubulin were used to probe blots to ensure equivalent protein expression for each protein. (B-C) Venn diagram overlap analysis was performed to determine the number of significantly regulated activated and repressed genes for both EF DBD and EF DBD+ in the HEK-293EBNA cells (N=3 biological replicates each). Significantly regulated genes were determined using a FDR cut-off of 0.05. (D-E) GSEA analysis comparing genes regulated by EF DBD+ as the rank-ordered gene list to a gene set of (D) activated genes by EF DBD (log(2)FC > 0, FDR < 0.05) or (E) repressed genes by EF DBD (log2(FC) < 0, FDR < 0.05).

**Supplementary Figure 5. Heatmap analysis of ATAC-signal at EWS/FLI-mutant bound activated and repressed genes.**

(A-B) Heatmaps depicting CUT&RUN and ATAC-sequencing signals, centered on the nearest transcriptional start site (TSS) of genes regulated by EF DBD+ only or both EF DBD+ and EF DBD. EWS/FLI-mediated activated genes are visualized in (A) and repressed genes in (B). Knock-down cells (KD; iEF + Empty Vector), EF DBD+ (iEF + EF DBD+), and EF DBD (iEF + EF DBD) were compared (scales for peak height are depicted below heatmaps). The log2(FC) of RNA expression for EF DBD+ and EF DBD (compared to KD) is pictured on the right with log2(FC) scale depicted below.

**Supplementary Figure 6. Flanking regions outside of the FLI DNA-binding domain are not crucial for the ability of FLI to bind DNA.**

(A-B) Coomassie blue staining was performed on SDS-PAGE gels to analyze FLI DBD+ ΔN (B) and FLI DBD+ ΔC (C) recombinant protein purification. Samples were taken during various stages of purification, including eluted fractions after ion exchange chromatography (IEC). (C-E) Fluorescence anisotropy was performed using 0-20 mM recombinant protein and 5 nM of fluorescein-labelled DNA duplex on the following DNA sequences: (C) ETS HA site, (D) 2xGGAA-μSat, and (E) 20xGGAA-μSat. Each measurement was completed with 2 biological replicates, with 3 technical replicates in each. (F) Binding dissociation constants (K_D_) of recombinant FLI proteins were determined from fluorescence anisotropy data and quantification recorded here.

**Supplementary Figure 7. Disruption of the fourth alpha-helix of the FLI DNA-binding domain results in loss of oncogenic transformation potential of EWS/FLI.**

(A) Protein schematics of 3xFLAG-tagged EWS/FLI cDNA constructs, including versions of EF DBD+ ΔN that have mutations in the fourth alpha-helix of the DNA-binding domain of FLI. EF DBD+ ΔN α-helix Mutant includes four amino acid substitutions and EF DBD+ ΔN α-helix Pro(line) Mutant includes five proline-specific amino acid substitutions, both of which are predicted to disrupt the alpha-helical structure of this region. (B) Representative qRT-PCR results depicting knock-down of endogenous EWS/FLI mRNA in the A673 knock-down/rescue system; completed using EWS/FLI-specific primers and normalized to RPL30 mRNA values for each sample. Data are presented as mean ± SEM (N= 1 biological replicate with 3 technical replicates). Asterisks indicate significant knock-down of EWS/FLI as compared to iEF + Empty Vector control cells (p-value < 0.05). (C) Western blot analysis of A673 knock-down/rescue samples: α-FLAG used to probe for expression of the cDNA constructs and α-tubulin used as loading control. (D) Representative soft agar assay results showing oncogenic transformation potential of EWS/FLI cDNA constructs in A673 knock-down/rescue cells. (E) Quantification of colonies in soft agar assays represented by mean ± SEM (N= 3 biological replicates with 2 technical replicates each). Asterisks indicate significant transformation over control cells (iEF + Empty Vector) (p-value < 0.05).

**Supplementary Figure 8. Homodimerization motif is dispensable for EWS/FLI-mediated oncogenic transformation.**

(A) Protein schematics of 3xFLAG-tagged (3F) EWS/FLI constructs: EF represents full-length EWS/FLI; EF DBD+ contains EWS fused to the DBD+ version of FLI; EF DBD+ F362A represents EWS fused to the DBD+ version of FLI with the phenylalanine (F) residue at residue 362 mutated to alanine (A). (B) Constructs were expressed in A673 cells using our knock-down/rescue system and Western blot analysis was used to determine efficient expression of these proteins using α-FLAG antibody for detection of EWS/FLI constructs and α-Tubulin as a loading control. (Samples labeled as X are not relevant to this current set of experiments.) (C) Representative soft agar assay results are depicted, including controls and experimental samples. (D) Soft agar assay colony formation quantification. Data is represented by mean ± SEM (N= 3 biological replicates with 2 technical replicates each). Asterisks indicate p-value < 0.005 as compared to samples with EWS/FLI knock-down (iEF + Empty Vector).

**Supplementary Table 1. Amino acids references for EWS/FLI of FLI recombinant protein-encoding constructs**

Amino acid residues composition of each EWS/FLI cDNA or FLI recombinant protein-encoding construct are denoted here, according to the residue number in native EWSR1 (NP_001156757.1), native FLI1 (NP_002008.2), or “type IV” EWS/FLI translocation (identified by May et al., 1993).

**Supplementary Table 2. Sequences for primers used in qRT-PCR experiments**

Forward and reverse primer sequences used for qRT-PCR experiments to determine levels of endogenous EWS/FLI or RPL30 mRNA.

**Supplementary Table 3. Fluorescein-labeled DNA-duplex oligonucleotides used for fluorescence anisotropy experiments**

Forward sequences of fluorescein-labeled DNA-duplex oligonucleotides used for fluorescence anisotropy experiments.
